# Near Infrared Fluorescence Imaging in Nano-Therapeutics and Photo-Thermal Evaluation

**DOI:** 10.3390/ijms18050924

**Published:** 2017-04-28

**Authors:** Mukti Vats, Sumit Kumar Mishra, Mahdieh Shojaei Baghini, Deepak S. Chauhan, Rohit Srivastava, Abhijit De

**Affiliations:** 1 Department of Biosciences and Bioengineering, Indian Institute of Technology Bombay, Powai, Mumbai 410210, India; muktivatsiitb@gmail.com (M.V.); deepakchauhan@iitb.ac.in (D.S.C.); rsrivasta@iitb.ac.in (R.S.); 2 Molecular Functional Imaging Laboratory, Advanced Centre for Treatment, Research and Education in Cancer (ACTREC), Tata Memorial Centre, Kharghar, Mumbai 410210, India; sumitmishra1089@gmail.com (S.K.M.); mahdieh.shojaei.baghini@gmail.com (M.S.B.)

**Keywords:** NIR fluorescence, molecular imaging, photothermal therapy, gold nanoparticle, cancer

## Abstract

The unresolved and paramount challenge in bio-imaging and targeted therapy is to clearly define and demarcate the physical margins of tumor tissue. The ability to outline the healthy vital tissues to be carefully navigated with transection while an intraoperative surgery procedure is performed sets up a necessary and under-researched goal. To achieve the aforementioned objectives, there is a need to optimize design considerations in order to not only obtain an effective imaging agent but to also achieve attributes like favorable water solubility, biocompatibility, high molecular brightness, and a tissue specific targeting approach. The emergence of near infra-red fluorescence (NIRF) light for tissue scale imaging owes to the provision of highly specific images of the target organ. The special characteristics of near infra-red window such as minimal auto-fluorescence, low light scattering, and absorption of biomolecules in tissue converge to form an attractive modality for cancer imaging. Imparting molecular fluorescence as an exogenous contrast agent is the most beneficial attribute of NIRF light as a clinical imaging technology. Additionally, many such agents also display therapeutic potentials as photo-thermal agents, thus meeting the dual purpose of imaging and therapy. Here, we primarily discuss molecular imaging and therapeutic potentials of two such classes of materials, i.e., inorganic NIR dyes and metallic gold nanoparticle based materials.

## 1. Introduction

Molecular imaging (MI) reveals biological information that is relevant for the clinical understanding of disease processes, and thus carries enormous relevance for patient care. As the goal is to obtain images directly related to the activity of a molecular process in the body, MI includes two- or three-dimensional imaging and quantification capacity, providing dynamic molecular information in space and over time from a living subject. Thus, quantification is a key element of MI data and its analysis, especially for drawing intra- and inter-subject comparisons. Imaging of cancer lesions for simultaneous localization of the site as well as obtaining functional information for oncogenic protein molecules is of great clinical significance. Another aspect where MI can play a greater role is surgical interventions, which remains the mainstay, at least in oncology, for most complicated indications in spite of the improvement in other medications. Here, a real-time method of visualization is an essential requirement to suffice the technical challenges faced at the surgery table.

The imaging community has pursued various avenues by translating spectral imaging modalities from extant preoperative techniques that include single photon emission computed tomography (SPECT) and positron emission tomography (PET) [1]. Both imaging modalities have been explored with great success, however, cost, accessibility, and use of ionizing radiation associated with these techniques imparts limitations on their real-time translation. Furthermore, nonspecific uptake leads to an elevated background, which makes deciphering the surgical field challenging, thus obviating potential benefits. Optical imaging is a very promising imaging technique; the major strength of it lies in its ability to provide biophysical and molecular functional information on disease process at highest sensitivity [2]. Additionally, the ability to use in a non-invasive setting, faster scan operation, relatively lower cost, and easy production of probes make it very attractive in mitigating the demand. However, the major obstacles of optical imaging are low depth penetration, poor spatial resolution, absolute quantification as well as development, validation, and approval of relevant imaging agents for human use. The emergence of near-infrared fluorescence (NIRF) based optical imaging covers such shortfalls and thus marks a very attractive advancement in the field of cancer imaging and therapy [3]. In combination with versatile fluorescent probes and sensitive detection equipment, this technique can be applied to image a wide variety of molecular entities in vivo and in real-time. This review provides in-depth information of molecular imaging applications of inorganic NIR fluorescent dyes and metallic gold nanoparticle based materials. Further therapeutic potentials of such materials with special reference to photothermal therapy (PTT) are also discussed.

## 2. Imaging and Photothermal Therapy Using NIR Fluorescence Molecule

### 2.1. The Near-Infrared Window

The use of near infrared wavelengths of light for imaging promises high sensitivity. The use of NIR (700–1000 nm) light for biomedical imaging is grounded in first principles, and is best understood in the context of photon propagation through living tissue and the signal to background ratio (SBR). An excitation photon typically travels through tissue to reach the fluorescent contrast agent, and has several possible fates depending on the tissue’s scatter, anisotropy (g), and refractive indices [1]. The photon emitted by the fluorophore is also subject to the same fates. Such properties of light absorption and scatter severely affect the determination of the spatial details of the photon source within a tissue environment. Further, when tissue absorbs light, there is a chance that some of its ingredients will emit fluorescence. Generally, the photon absorbance of a particular tissue or organ is the sum of all absorbing components present. In living, non-pigmented tissue, the major NIR absorbers are water, lipids, oxyhemoglobin, and de-oxyhemoglobin, with the absolute value of μ_a_ depending on the molar concentration of each component [4,5]. Thus, in addition to photon absorption attenuation, tissue “autofluorescence” can also severely limit SBR. In a “typical” tissue, having 8% blood volume and 29% lipid content, the dominant absorber is hemoglobin, accounting for 39–64% of total absorbance at NIR wavelengths [5]. High tissue autofluorescence precludes the use of the visible range of light below 650 nm for most in vivo imaging applications, and NIR light reduces this burden by drastically reducing fluorescence background in the tissue environment. The opportunity for high SBR paired with cost-effective lasers, improved detectors, and the inherent innocuous nature of NIR light makes it a promising technology for further development [6].

### 2.2. Requisite Design Parameters of Imaging Probes

The NIR imaging agents must be designed satisfying a typical set of parameters which are requisite for its success [7]. Many classes of known fluorescent structures have been used successfully, which encompass three unique classes: (i) the small molecule fluorophores (the most studied class), such as cyanine, porphyrin based fluorophores, metal complexes, xanthene dyes, squaraine, rotaxanes, and phenothiazine-based fluorophores; (ii) synthetic nanoparticles such as quantum dots; and (iii) biologics such as variants of red fluorescent protein [5,8]. All of these representative agents must be tailored to achieve sufficient stability, specificity, and safety for use in living bodies. These properties are highly important for future clinical translation and must be maintained throughout the developmental process. The sensitivity, specificity, pharmacokinetics, delivery, and toxicity depend highly on the targeting method used and the overall chemical composition of the contrast agent. Where the stability is a determining factor to in vivo success, the chemical bonds and moieties present only limit the choices available for modification, which does not directly influence the tissue-specific imaging characteristics.

### 2.3. Methods for Obtaining Tissue Specific Imaging

Tissue specificity is determined by a simple comparison of the signal strength gathered in the targeted tissue to the signal in the surrounding area. This ratio plays a fundamental yet crucial role in the imaging of small and otherwise undetectable tissues. For example, when contrast agents fail to display high tissue specificity resulting in low SBR, small tumors or occult metastases would remain invisible, and the imaging procedure would not afford meaningful guidance [5]. Overcoming these obstacles is challenging and has been the focus of a recent research thrust with various research laboratories engineering contrast agents that exploit innate biological/physiological systems to achieve an optimal SBR.

One predominant and natural strategy which relies on the bio-distribution of a contrast agent to achieve tumor-selective imaging is the passive targeting via enhanced permeability and retention effect (EPR). For example, the leaky vasculatures of tumors frequently allow larger molecules (100–400 nm size range) to enter the tumor bed compared with the more discriminating healthy tissue. In normal tissue, the gap junctions between the endothelial cells forming the wall of vasculature ranges between 2–10 nm and thus molecules exhibiting a particular size, hydrophobicity, or molecular recognition moiety may only enter the supply area. This preferential accumulation ability of molecules in the tumor bed offers a natural attainment of tissue-specific imaging, at least in the majority of cancer types.

The active approach involves numerous targeting ligand-fluorophore tethering approaches, which have been explored with varying degrees of success. Requisite factors in the in vivo performance of tethered fluorophores are the targeting ligand, the isolating linker, and the dual-purposed fluorophore with effector and balancing domains. Successful implementation of this approach requires several specific engineering hurdles. An engineered NIR fluorophore being synthetically tethered to a targeting moiety, such as a surface biomarker, has been effectively exploited for homing contrast agents directly to diseased tissues [9]. Perhaps the most crucial aspect for the success of a targeted probe design is the choice and overall binding affinity of the targeting ligand. Through direct covalent conjugation to the effector domain, this ligand may target and bind surface molecules or overexpressed receptors on the cell surface. Other factors include the molecular weight and size of the contrast agent, which must also be considered while designing an active-targeting agent. For the effector domain, the optical profile, specifically the Stokes shift, extinction coefficient, and quantum yield is highly dependent on the rigidity of the core fluorophore structure, specific modifications to the conjugated system, and solvent-fluorophore effects. The importance of optimizing the physicochemical, structural, and dynamic properties of the isolating linker cannot be underestimated as well. This is a very rapid process that offers the potential for high SBR and near complete elimination from the background associated with reduced nonspecific binding. However, if a specific tumor fails to express a high amount of the surface molecule, SBR is significantly lowered.

Current research is also actively pursuing stimuli responsive design of probes. Once injected, such molecules display diminished fluorescence intensity while roaming through the body and only get activated at a target site when exposed to the specific stimulus associated with the tissue microenvironment (i.e., pH, metabolite concentration, enzyme, redox potential in hypoxic cells, etc.) [10]. This can be a slow process with non-specific distribution of the molecules. However, ideally the molecules distributed non-specifically are never activated; thus, this strategy can provide a very low background signal and an overall high SBR. A variant to such an approach is, of course, the use of materials which change properties under an externally triggered signal, such as thermosensitive, photosensitive, or even ultrasound sensitive materials [11].

## 3. Emergent Applications of NIR Fluorescent Probes for Imaging

### 3.1. Nanoparticle-Based Bio-Conjugates

The molecular design of a nanoparticle-based contrast agent must feature four major components: targeting ligand, isolating linker, effector domain, and balancing domain [12]. Such approaches have been utilized extensively in the design of nanoparticle-based contrast agents for optical image-guided disease monitoring [13,14] and surgery guidance, of which in vivo performance depends strongly on their molecular design and physiochemical and optical properties. Recent advancements in nanoparticle-based imaging suggest high promise in the future; currently, however, the intrinsic character of nanoparticles does not readily lend itself to biological compatibility. Owing to this principle, rapid clinical translation is uncommon in the nanoparticle space [15]. Though nanoparticles are not preferred by regulatory agencies, small molecule imaging agents offer a unique and appealing alternative with respect to the ability to synthesize a single chemical entity with high reproducibility and purity. With the Food and Drug Administration (FDA)-approved NIRF dye indocyanine green (ICG), it is likely that such nanoscale molecular imaging agents will play a big role in developing clinically relevant probes for tissue-specific imaging and cover robust and cutting edge research on developing small molecule contrast agents.

### 3.2. Small Molecule-Based Bio-Conjugates

Similar to the first model, there is a targeting ligand that serves as the homing beacon to direct the imaging agent to the tissue of interest; however, the effector domain must remain either comparatively small (against the targeting ligand) or biologically silent through the synthetic incorporation of a balancing domain within the structure of the fluorophore. These two design approaches are not equal, since reducing the effector domain size effectively limits the aromatic system, resulting in non-NIR absorbance and fluorescence wavelengths. The even more challenging part is the dual channel imaging capability, which requires targeting healthy tissue, thus necessitating the engineering of additional fluorophores that exhibit native tissue selectivity. Cellular surface receptors and subcellular targeting domains for native healthy tissue remain scarcely known within the literature; therefore, a tethered approach would not be an obvious choice for obtaining tissue-specific contrast agents for normal tissue. Many of the fluorophores described to date have one or more detrimental shortcomings ranging from limited chemical and optical stability to insufficient fluorescence quantum yield in serum or high background signal in vivo arising from nonspecific binding to extracellular proteins. Such chemical structures have been extensively modified for decades with only minor improvements to tissue affinity and background reduction. Choi et al. explored the importance of judiciously applying a charge in the engineering of fluorophores for maximizing targeting efficacy by carefully incorporating zwitterionic character into a heptamethine cyanine chromophore. The final fluorophore, named as ZW800-1, results in minimal interactions with serum protein, thus gaining the unique ability to effectively target the corresponding receptor [16]. In fact, when the commercial alternative, Cy5.5, and the ZW800-1 analogs are modified with cRGD, these authors observed unparalleled tumor targeting with low nonspecific background.

## 4. Emergent Therapeutic Applications of NIR Fluorophores in Cancer Research

### 4.1. Drug Delivery

Chemotherapy has been widely applied to maximize therapeutic outcomes in cancer treatments. However, most cytotoxic drugs lack the ability of specific accumulation in tumors. In addition, various side effects may occur during the course of chemotherapy. These remain major impediments to the treatment of malignancies. Thus, novel platforms for targeted drug delivery that are safe and effective in vivo are highly desirable. Effective delivery of chemical drugs to tumor sites is particularly appealing for the enhancement of the tumor-killing effect and the reduction of systemic toxicities. It was revealed that drug-loaded nanoparticle systems accumulate in tumors through the EPR effect, which increases drug bioavailability and prolongs the exposure to therapeutic agents. Rapid uptake and retention of polymer conjugates in the lymphatic system have also been observed with low toxicity.

Mieszawska et al. presented a highly complex and multifunctional hybrid polymer lipid NP platform that incorporated diagnostic nano-sized crystals and two therapeutic drugs, the anti-angiogenic drug sorafenib and the cytotoxic drug doxorubicin, for combined cancer therapy [17]. The prepared NPs accumulated at the tumor sites and prevented angiogenesis, leading to cancer cell death. NIR irradiation of a light-sensitive amphiphilic co-polymer cleaved the capote-containing micelles and released cytotoxic *O*-nitrosobenzaldehyde that could damage the surrounding tissues [9]. Turner et al. successfully constructed various temperature-sensitive NIRF mixtures to realize efficient drug delivery [18]. The thermosensitive liposome composites were stable at 37 °C, while burst releases of encapsulated drugs occurred at 40–42 °C.

Currently, antibodies against biomarkers and therapeutic targets for cancer have already been developed. The crosslinking of monoclonal antibodies and NIRF dyes has also been applied for selective cancer theranostics, such as cutaneous, breast, ovarian, gastric, and prostate cancer [18]. This controlled drug delivery may potentially address the described limitations of the aforementioned chemotherapy.

### 4.2. Photo Dynamic Therapy (PDT)

PDT utilizes light irradiation that is enhanced by photosensitizers to exert therapeutic effects in cancer tissues. When excited by light of a certain wavelength, photosensitizers facilitate the generation of cytotoxic free radicals. These products affect tumor growth by destroying the abnormal neo-vasculature directly. They also initiate an inflammatory microenvironment that leads to cancer cell death. The first approved photosensitizer, Photofrin, is a composite of oligomeric porphyrins that has been applied for the treatment of lung, esophagus cancer, etc. In 1993, Photofrin was applied for the first time as a PDT agent to treat bladder tumors [19,20]. It is noteworthy that current PDT using Photofrin exhibits many drawbacks that limit wide clinical application, such as low deep-tissue penetration, limited tumor specificity, and unwanted localization, especially in the skin, which leads to skin photosensitivity after sunlight exposure. Various NIRF sensitizers have been tested for use as PDT agents, such as classical cyanines, squaraines, porphyrins, and phthalocyanines and their derivatives [21]. However, screening new photosensitizers to overcome these basic limitations is imperative for expanding PDT applications.

### 4.3. Photo Immune Therapy (PIT)

PIT is based on cancer-targeted therapy that can selectively monitor and destroy cancer cells. Nakajima et al. developed a NIRF probe for PIT by linking a phthalocyanine dye IR700 and a monoclonal antibody [22]. When exposed to NIR light, the conjugates that had been accumulated in the target sites induced highly specific tumoricidal activities. Selective binding avoided unnecessary injury to normal tissues. It was revealed that IR700 eventually accumulated in lysosomes. After exposure to a threshold intensity of NIR light, the conjugates immediately disrupted the outer cell membrane and lysosomes. Furthermore, repeated application of NIRF dyes was an effective strategy for cancer therapy without severe side effects; complete pathological remission might even be achieved through this approach.

### 4.4. Photo Thermal Therapy (PTT)

**Figure 1:**
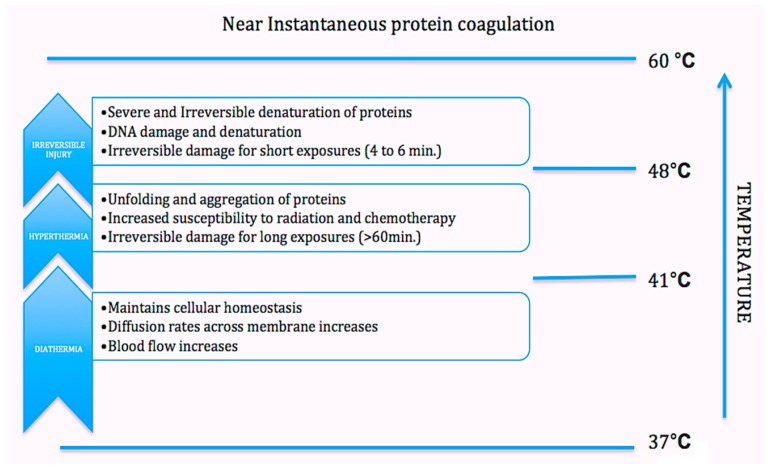
Schematic illustrating biological effects linked to stage-wise thermal increments with corresponding temperature rise.

PTT, a non-invasive treatment effective for treating many diseases, has been extensively investigated in cancer as well. Minimally invasive, non-toxic laser thermal therapy engendered by NPs or drugs is among the most promising technologies to arrest expansion of cancerous growths with minimal morbidity and reduced toxicity. Laser-absorbing agents or dyes are used to increase laser-induced thermal damage in the tumor (Figure 1).

**Figure 2:**
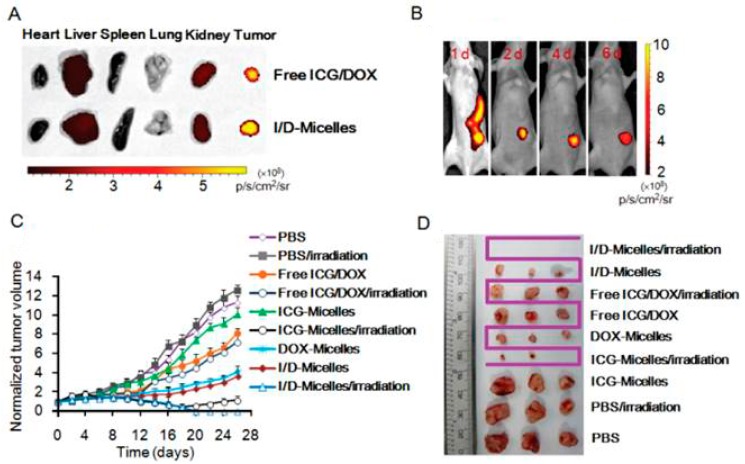
Preclinical test results showing ex vivo and in vivo imaging and photothermal effect measurement of indocyanine green (ICG)-micelle with or without doxorubicin (DOX) drug loaded nanoparticles. (**A**) Ex vivo imaging of ICG from free ICG/DOX and ICG/DOX-micelles in heart, liver, spleen, lung, kidney, and tumor of the mice at 24 h post-injection at the dose of 7.5 mg/kg ICG/DOX, respectively; (**B**) In vivo near-infrared fluorescence (NIRF) imaging of the mice bearing A549 tumor injected with I/D-micelles at the dose of 7.5 mg/kg ICG/DOX at 1, 2, 4, and 6 days post-injection, respectively; (**C**) Tumor growth inhibition profiles of the mice bearing A549 tumor injected with various formulations; (**D**) Photographic view of tumors extracted from the mice bearing A549 tumor at the end of the experiment. Figure adapted from [25].

The accumulation of NIRF probes in tumor sites drastically increases the efficiency of PTT through effective conversion of light energy into heat. It has been demonstrated that ICG promotes the absorption of NIR laser light delivered by a diode laser, inducing more thermal damage to solid tumors after laser irradiation compared to laser alone [23]. In addition, local hyperthermia greatly enhances the delivery of ICG to the tumor site and interstice, thereby allowing a greater thermal ablation effect on the tumor cells, vasculature, and surrounding tumor matrix to induce tumor regression. Hyperthermic changes by using ICG (37 to 43 °C within 1 min) has also been proven to be a safe approach to overcome multidrug resistance [24]. Due to limitations such as poor photostability, self-aggregation, rapid elimination from the body, and lack of target specificity, ICG is usually encapsulated into the core of a polymeric micelle and shown for its potential application in tumor photothermal therapy (Figure 2). Apart from ICG, cyanine dye IR820 has optical and thermal generation properties as well. It may be an alternative to ICG with greater stability, longer image collection times, and more predictable peak locations. Other types of NIRF dyes, such as phthalocyanine-aggregated pluronic NPs and IR780-loaded NPs are also constructed as novel agents for PTT and/or fractionated PTT for clinical use [18]. Some of these probes have obtained promising results in preliminary experiments. Researchers will continuously focus on developing appropriate NIRF platforms for photothermal applications.

## 5. Imaging and Photo-Thermal Therapy Using Gold Nanoparticles

### 5.1. Physical and Optical Properties of Gold Nanoparticles

Gold nanoparticles possess unique physico-chemical and optical properties, primarily governed by the size and shape of the gold particle. Gold nanoparticles (with a size range of 2–100 nm) differ from bulk gold due to the presence of a phenomenon called surface plasmon resonance (SPR) [26]. SPR occurs as a result of the collective coherent oscillation of the conduction band electrons at the nanoparticles surface as a result of absorption of the resonant incident light. This interaction leads to a loss in the energy of electromagnetic radiation contributed by absorption and scattering processes [27]. Changing the size of a gold particle alters the SPR, which leads to a corresponding change in all radiative and non-radiative processes associated with it. For example, gold nanoparticles that are up to 20 nm in size have higher extinction efficiency mostly due to absorption [28]. Hence, smaller nanoparticles can efficiently adsorb light and convert it into heat, sufficient to destroy tissues and cells, finding application in photo-thermal therapy (PTT). On the other hand, larger extinction cross sections for particles above 50 nm is mostly due to scattering [29]. Thus, as a result of higher scattering efficiency, larger nanoparticles are harnessed for tissue imaging. Surface plasmon of gold is also determined by the shape of the gold particle; gold nanospheres have one SPR, whereas gold nanorods have one longitudinal and another transverse resonating plasmon mode of different wavelengths. This dependence of SPR on the shape, size, and composition of nanoparticles can be exploited for surface enhanced Raman-scattering (SERS) based imaging of tissues.

SERS is a surface sensitive technique that enhances Raman scattering by molecules adsorbed onto rough metal surfaces or by nanostructures such as copper, silver, and gold [30]. SERS amplification with gold nanoparticle may occur by either electromagnetic enhancement or chemical enhancement. As a consequence of SPR, the electromagnetic field near the gold nanoparticle surface is enhanced, and this enhancement increases the Raman scattering of the adjacent molecules [31]. Thus, electromagnetic enhancement by gold plasmon accounts for increases of Raman scattering up to the order of 10^6^–10^7^ [32]. Simultaneously, gold particles conjugated to different Raman sensitive dyes also enhance the SERS associated with gold. However, this increase in Raman scattering is only up to the order of 10^2^ and happens because the molecule adsorbed on the surface of the nanoparticle leads to a change of its polarizability [32].

Gold nanoparticles also possess a negligible luminescence signature with a quantum yield of 10^4^–10^5^ times more than bulk gold [29,33]. The luminescence efficiency of gold particles can be further enhanced by surface adsorption of organic dyes as well as by increasing the surface roughness. Surface plasmon resonance also attributes luminescence enhancement to the gold particle [33,34]. Apart from luminescence, resonant scattering of light by gold nanoparticles upon excitation of the surface plasmon also imparts it an inherent fluorescence, which even under continuous light illumination is immune to photobleaching [35]. Alternatively, metallic nanoparticles like gold, due to their high polarizability, display prominent interactions with fluorescent chromophores in the vicinity. The plasmonic field generated by the gold particle either enhances or quenches the fluorescence quantum yield of the fluorophore, and this effect generally operates at a distance of around 10 nm [36]. Enhancement of the fluorescence, especially of low quantum yield fluorophore due to the nano-lensing effect of the nanoparticle, offers greater sensitivity and high SBR that is well suited for molecular imaging. Thus, as a result of shape and size dependent change in the properties of nano-sized gold particles, they can be fine-tuned to absorb light in the NIR region (where the interference through the biological tissues and fluids is minimum) and this phenomenon can be efficiently utilized in NIR based cancer theranostics utilizing gold nanoparticles.

Photo-thermal therapy is a non-invasive therapeutic mode and a form of hyperthermia in which NIR light energy absorbed by plasmonic material is converted into heat [37]. A wide range of hyperthermic nanomaterials such as graphene, CuS nanoparticles, carbon nanotubes, silver based nano-constructs, Pd nanoparticles, as well as Au nanoparticles have gained significant attention in PTT [38]. However, the feasibility of harnessing plasmonic gold nanoparticles for heat induced ablation of cancerous cells has been very well illustrated by different groups. Lin et al. first demonstrated the efficacy of gold nanoparticles for PTT mediated extirpation of cells by using anti-CD8 labelled gold nanoparticle for selective targeting and destruction of T cells [39]. Highlighting the efficacy of nanoparticles as an effective photothermal agent, heat induced killing of bacterial cells using gold nanoparticles was also demonstrated [40]. However, the use of plasmonic gold nanoparticles for selective targeting and ablation of cancer cells was first elaborated by El Sayed et al. in the year 2006. Using benign (HaCaT) and malignant (HSC and HOC) oral squamous carcinoma cell lines, it was shown that anti-EGFR conjugated gold nanoparticles were able to destroy cancer cells when irradiated with a 514 nm laser for 4 min [28]. These initial studies accentuate the use of gold nanoparticles as a novel class of agents that are well suited for hyperthermia induced cell killing.

**Figure 3:**
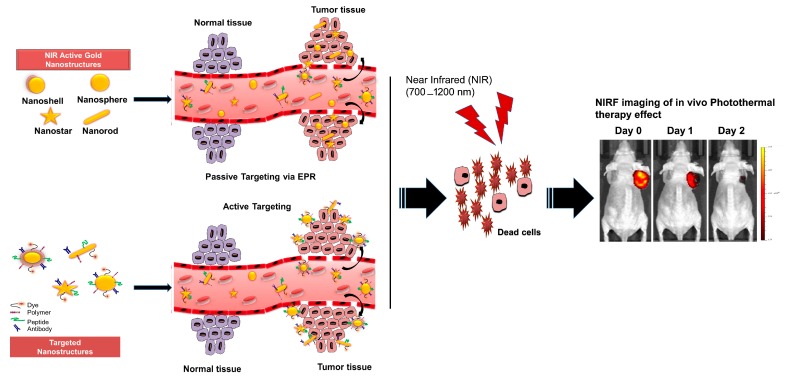
Schematic showing gold nanoparticle based near infra-red (NIR) mediated image guided photo-thermal therapy. EPR, enhanced permeability and retention effect.

Gold nanoparticles have been recognized as the best plasmonic material [41] and have gained significant attention in the field of cancer research, especially in cancer theranostics. Gold nanoparticles (AuNPs) can be formulated in different shapes such as nanospheres, nanorods, nanoshells, nanostars, and nanocages because they exhibit excellent size and shape tunability and physiochemical properties [42]. Although AuNPs illustrate different shapes, all possess certain unique properties such as biological inertness and biocompatibility, the ability to absorb high energy X-rays, and they can be fine-tuned to absorb light in the near infra-red region (700–1200 nm) due to inherent SPR [43]. Considerable progress has been made towards the development of NIRF sensitive nanoparticles for efficient cancer imaging, identification of residual cells during surgery, and for locating the surgical margins [44]. Recently, NIR coupled two-photon luminescence imaging (TPL) and SERS have been efficiently utilized for cancer imaging by conjugating gold nanoparticles to fluorescence reporter molecules. This is associated with increased depth of tissue penetration, reduced photo-toxicity, and efficient light detection [45], allowing enhanced tissue contrast as compared to conventional magnetic resonance imaging (MRI) or computed tomography (CT) based imaging. Despite the elaborate advantages of utilizing gold nanoparticle in cancer biology, its use in image guided PTT still requires further development. This is readily achievable, as gold is associated with straightforward chemistry [42], has a high surface area to volume ratio, and provides ease of surface modifications [46]. This allows for efficient loading of a large amount of cargo including fluorescent dyes, chemotherapeutic agents, proteins, and peptides to the nanoparticles [46]. Ultimately, these surface modifications are associated with selective tumor targeting and accumulation, increased biodistribution and biocompatibility, superior contrast enhancing properties, and reduced toxicity. Thus, surface functionalization of gold nanoparticles annotates it as a single multifunctional platform for NIR based cancer imaging and photothermal therapy (Figure 3).

#### 5.1.1. Gold Nanostars

Recently, gold nanostars have gained attention as a contrast enhancing agent as well as a PTT agent because of more NIR light absorbing capability and low toxicity. Gold nanostars possess multiple thin branches, and hence show tip-enhanced plasmonic properties [47] and display comparatively low radiative light scattering as compared to other gold nanostructures [48]. A wide range of surface functionalization strategies applied on these nanoparticles popularize their use for both in vivo imaging and PTT.

Gu et al. synthesized multifunctional gold nanostars (Au-cRGD-MPA and Au-cRGD-DOX). NIR irradiation of Au-cRGD-MPA nanoparticle treated breast cancer cells and xenografted mouse revealed peak fluorescence intensity of MPA (hydrophilic indocyanine green derivative) probe (8 h in vitro and 12 h in vivo post-treatment) allowing efficient tumor imaging. Au-cRGD-DOX nanoparticle treatment in vivo reflected 90% cancer cell death and 100% animal survival 4 weeks past laser treatment. Ex vivo histological examination also confirmed hyperthermic killing of tumor cells in the nanoparticle treated group [49]. The conjugation of Raman reporter with gold nanoparticles provides a novel probe for in vivo biosensing and therapy. Vo-Dinh et al. synthesized PEGylated-Au nanostars (GNS) conjugated with Raman reporter p-mercaptobenzoic acid (pMBA) for optical imaging using surface enhanced Raman scattering (SERS) as well as PTT. Photothermal efficacy measurement of GNS in vitro showed laser power dependent temperature increments after NIR laser irradiation. Infrared thermal imaging of mouse surface also showed hyperthermia, with tumor surface temperature increasing up to 50 °C, which is sufficient to kill tumor cells [47]. Kohane et al. generated gold nanostar with a shell of metal-drug coordination polymer (AuNS@CP) for combined chemotherapy, imaging, and photothermal therapy. Thermal imaging showed increased tumor temperature sufficient to irreversibly damage cancer cells. The CP shell of the modified nanostar (gadolinium + gemcitabine) allowed for MRI and chemotherapy. However, gold nanostar core allowed for heat mediated killing and photoluminescence imaging of tumor cells [50]. Shi et al. generated multifunctional gold nanostar based nanocomposites (MGSNs) for bimodal NIR based imaging and PTT. In vitro experiments with MGSNs treated breast cancer cells (MDA-MB-231) revealed more than 90% cell death after irradiation with 808 nm NIR laser. Infrared thermal images of tumor bearing mice post-nanoparticle plus NIR laser treatment showed complete tumor regression after 2 days. The resultant MGSNs serve as excellent agents for simultaneous SRES imaging and photothermal therapy of cancer tumors [51].

#### 5.1.2. Gold Nanospheres

Photo-thermal therapy using gold nanospheres was first demonstrated by Lin and co-workers [37]. Because of their small size, ease of ligands conjugation, and fast synthesis, gold nanospheres serve as attractive biological platforms for NIR coupled tumor imaging and irreversible cellular destruction through PTT [52]. West et al. investigated in vitro NIR PTT using anti-HER2-conjugated gold-gold sulfide nanoparticles (GGS-NPs). The GGS-NPs bind specifically to the surface of malignant breast cancer cells (SK-BR-3) that overexpressed HER2. In vitro experiments with GGS-NPs showed sharp TPL signals from breast carcinoma cells after NIR laser irradiation. Increased heat generation and subsequent ablation of malignant cancer cells were also seen at the laser power of 50 mW. This study indicated that gold nanoparticles have an inherent TPL property, and can be functionalized for selective targeting, visualization, and hyperthermia mediated extirpation of cancer cells [53]. On the contrary, Chao et al. reported a study of metal coupling to gold nanoparticles generating hybrid Ru2@AuNPs. Modified AuNPs treated HeLa cells showed enhanced red two photon luminescence signals, as compared to only Ru2 treated cells, which were reasonable for tumor imaging. Ru2@AuNPs treated tumor cells also displayed efficient nanoparticle uptake. Intratumoral injection of nanoparticles in xenografted nude mice after NIR laser irradiation showed almost 100% reductions in tumor volumes. Histological analysis also displayed significant necrotic regions in the tumor tissues, indicating efficient hyperthermic killing by surface modified nanoparticles [54]. In contrast to metal functionalized nanoparticles, Tian et al. synthesized dual modality gold nanoparticles (Au@MSNs-ICGs) for tumor imaging and ablation by harnessing NIR responsive ICG dye. Treatment of HepG2-Fluc cells with the nanoconstruct allowed visualization of tumor cells and the assessment of reduced cell viability after NIR laser irradiation in vitro. Xenografted nude mice treated with modified gold nanospheres after NIR laser exposure displayed a significant reduction in the tumor volume and facilitated distinct visualization of tumor tissue in vivo. The ICG loaded mesoporous silica core of the nanoparticles not only allowed potential tumor imaging ability, but also increased the heating efficiency of the gold metal, thus helping to facilitate NIR based image-guided PTT [55]. Kang et al. conjugated NIR fluorophore cyptate to hollow gold nanospheres (HGNs) by a urokinase-type plasminogen activator (uPA a breast cancer enzyme) enzyme- substrate motif. A surface functionalized nanoconstruct allowed for selective visualization of tumors as a result of increased fluorescence by cleavage of fluorophore specifically in cancer cells. HGNs also allowed for the characterization of tumor nature (metastatic, invasive, etc.) and showed hyperthermic killing of cancer cells [56]. Recently we have demonstrated the efficacy of thermo-labile liposome based gold nanospheres (LiposAu NPs) made for PTT. This design demonstrated excellent photothermic effect and irreversible photothermal destruction of tumor cells both in vitro and in vivo. The biocompatible liposome core of the nanoparticle endowed it excellent biodegradable capacity that allowed efficient body clearance of the gold via hepato-biliary and renal route [57,58].

#### 5.1.3. Gold Nanoshells (AuNSs)

Halas and co-workers were the first to develop gold nanoshells, which are composed of very thin outer metallic layers of gold and dielectric cores made up of silica [59]. The shell thickness and core diameter can be modulated to make them absorb light in the NIR region [26] for hyperthermia mediated killing of tumor cells [59]. Since their development, different surface modifications have been applied to these particles, making them better agents for NIR imaging and PTT of tumor cells.

West et al. synthesized polyethylene glycol (PEG) conjugated gold nanoshells by combining nanoshells with PEG-SH [59]. An in vivo study performed on (CT26.WT murine colon carcinoma cells) xenograft mice showed complete tumor regression 10 days post (laser + nanoshells) treatment. The mice remained healthy and tumor free even after 90 days post-treatment, demonstrating the efficacy of PEGylated gold nanoshells mediated tumor ablation [59]. Drezek et al. fabricated a novel immune-targeted nanoshells based platform and conjugated them to anti-HER2 antibody for targeting HER2 positive breast cancer cells. The nanocomplex integrated scattering contrast for imaging and photothermal heat generation property sufficient to kill cancerous tumors. This was the first report highlighting the coupling of a bio-imaging application to a cancer therapeutic using modified nanoparticles [60]. Mosquera et al. reported the use of PLGA/DOXO-core Au-branched shell nanostructures (BGNSHs) functionalized with human serum albumin/indocyanine green/folic acid (HAS-ICG-FA) as a tri-modal (BGNSH-HAS-ICG-FA) nano-theranostic platform. Tumor cells incubated with BGNSH-HAS-FA nano-platform after NIR irradiation exhibited reduced cell viability. However, the cell death increased significantly in BGNSH-HAS-ICG-FA treated tumor cells after NIR light exposure (808 nm, 2 W/cm^2^) because of the synergistic photothermal effect from gold metal and the NIR responsive ICG dye [61]. Joshi et al. used magneto-fluorescent theranostic gold nanoshells (TGNS) for selectively targeting pancreatic cancer cells. Irreversible photothermal destruction of the adenocarcinoma cells (AsPC 1) treated with nano-complex was seen after NIR irradiation in vitro. The TGNS encapsulate indocyanine green dye and iron oxide as contrast agents for fluorescence and MRI, respectively, while anti-NGAL antibody conjugation to TGNS facilitated selective targeting of pancreatic tumors [62].

#### 5.1.4. Gold Nanorods

Synthesis of gold nanorod was first reported by Wang and co-workers [37]. However, PTT effects using this material were unknown until 2006, which were first demonstrated by El Sayed et al. [27]. Nanorods exhibit strong photothermal effects because of the presence of two plasmon: one longitudinal and the other transverse [63] and possess unique polarization properties [41].

Chen et al. investigated the performance of chitosan oligosaccharide modified gold nanorods (CO-GNRs) for image-guided photothermal therapy. They incubated human oral adeno-squamous carcinoma cell line (CAL 27) with modified gold nanorod and observed GNRs selectively in malignant cells because of conjugation of GNRs with anti-EGFR antibody. Photothermal imaging of CAL 27 xenografted tumors displayed significant temperature increments (~up to 71 °C) specifically at the tumor microenvironment after NIR irradiation, which is well suited for PTT. Modified nanoparticles also did not display any associated toxic effect in vivo [64]. Cheng et al. reported a study of ultrasmall (10 nm) dendrimer-stabilized gold nanorods (DSAuNRs) for photothermal destruction of cancer cells. Lung adenocarcinoma cells treated with modified AuNRs after NIR exposure (808 nm, 3.6 W/cm^2^) exhibited nearly 100% cell death in vitro. Thermographs of xenografted nude mice intravenously injected with AuNRs displayed time-elapsed temperature increments from the tumor sites. A significant reduction in tumor volume following NIR treatment was also observed in vivo [65]. Cui et al. fabricated AuNRs loaded onto human induced pluripotent stem cells (AuNRs-iPS). NIR irradiation of human gastric cancer cells after AuNRs-iPS treatment resulted in significant reduction in tumor volume as a result of apoptosis induced by thermal effects. Thermal imaging also showed enhanced temperature generation in the tumor site that was sufficient to destroy malignant cells. Thus, iPS functionalized gold nanorods provide a novel platform for effective tumor targeted delivery and enhanced PTT [66]. Chen et al. demonstrated the efficacy of tLyp-1 peptide-functionalized, indocyanine green (ICG)-containing mesoporous silica-coated GNRs (I-TMSG) in NIR based PTT and tumor imaging. Infrared thermal imaging illustrated significant temperature increments of the GNRs dispersed solution which were suitable for PTT. Nanoparticle treated MDA-MB 231 cells also displayed significant cell death following NIR laser irradiation (785 nm, 3 W/cm^2^ for 3 min) [67].

### 5.2. Molecular Imaging Methodologies Using NIRF Signature

Recent advances in optical technology have taken fluorescent imaging beyond the standard two-dimensional (2D) epifluorescence imaging into the realm of three-dimensional (3D) space. Concurrent to the improvement in NIRF probe design, significant developments in sensitive instrumentation and fluorescence molecular tomography (FMT) have contributed to the effective measurement of localization and accurate quantification of probe accumulation in 3D volume within deep tissue organs [68,69,70]. This requires the trans-illumination of subjects (i.e., passing the light through the animals) rather than the standard surface illumination used in epifluorescence assessment. This advance brought by fluorescence tomography is accompanied by the need for extra care in performing imaging. Experimental animals must be prepared for trans-illumination imaging by the removal of hair, must be properly injected with imaging agents for optimal delivery to the imaging sites and minimization of artefacts, and scans must be performed under optimal conditions and settings. When performed properly, the pairing of powerful, deep tissue FMT imaging with appropriate near infrared (NIR) imaging agents allows the detection and quantification of important biological processes, such as cellular protease activity, vascular leak, and receptor upregulation, by accurately reconstructing the in vivo distribution of systemically-injected NIR imaging agents [68]. The ability to use fluorescent imaging agents that detect and quantify a variety of biological activities is already expanding the horizons of pre-clinical research and drug development.

Various Bayesian methods have been well developed for 3D reconstruction in NIRF (near infrared fluorescence) imaging and were used predominantly until 2003. For example, a study has shown the development of APPRIZE (automatic progressive parameter-reducing inverse zonation and estimation) for the reconstruction of fluorophore absorption by a 3D tissue phantom [71].

## 6. 3D Tomography

### 6.1. Fluorescence Imaging Tomography

In recent times, 3D in vivo tomography approaches (for small animal models) include PTOCT (Photothermal Optical Coherence Tomography) [72] and PAOCT (Photoacoustic Optical Coherence Tomography) [73]. These approaches are used for tissue imaging and, with the aid of external magnetic field(s), are fundamental in the development of tumor models, MMOCT (magneto-motive OCT) [73]. Ever since, a variety of instruments for 3D FLIT (fluorescence imaging tomography) have been developed, the most notable of which is the IVIS Spectrum in vivo Imaging System™ (PerkinElmer). Based upon our experiences in 3D in vivo imaging experimentation using this system, it has proven to be utilitarian in finding the following parameters.

(i) Depth of the tumor (axial visualization).
(ii) Localization of the host organ to which the tumor is closest. By capturing the tumor illumination, it can be used to find its proximity to vital organs, which might not be visible in the planer image.
(iii) The axes along which the tumor has maximum growth. This is necessary for determining the angle of injection (if intratumoral).
(iv) Centre of mass (COM) of the tumor can be used for spatio-temporal simulations [74].

**Figure 4:**
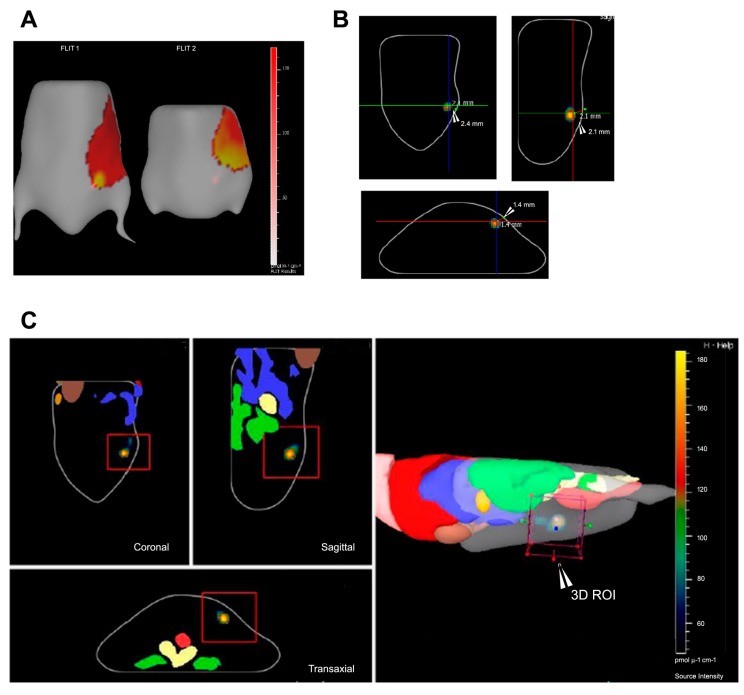
2D vs. 3D imaging of NIRF probe in mouse xenograft. (**A**) 2D planer image showing NIRF signal at source (tumor); (**B**) 3D coronal, sagittal, and transaxial image views with slice plane optimization, showing the center of mass of the tumor from the surface of the body; (**C**) Corresponding coronal, sagittal, and transaxial image views with the 3D region of interest (ROI) marked on reconstructed mouse image with overlapping organ atlas.

The software platforms associated with the current widely used optical imaging device comprises of two major types of tumor visualization, viz. voxelated view and source (tumor)-surface view. The latter can be used for geometric visualization of the fluorescent source and not for the depth calculation and quantification. The former, which is more commonly used, involves the construction of a 3D ROI (Region of Interest) around the illumination source (i.e., tumor) to find the total fluorescence yield of the selected voxels (pmol·M^−1^·cm^−1^) (Figure 4).

Removal of the tissue auto-fluorescence accounts for the increase in SBR ratio, which increases the overall efficiency of tumor reconstruction. Trans-fluorescence illumination mode for deep tissue 3D analysis is being used as opposed to conventional microscopy of the tumor. The depth is decided by choice of the emission and excitation filters used, which in turn depends on the choice of NIRF probe [75]. Each filter serves as a bandpass filter with the fluorophore absorption peak located at the peak of the bandpass filter [76]. Due to the availability of variegated fluorescent probes, multi-dye imaging techniques are now being used for bio-imaging (they can be easily extrapolated to 3D in vivo imaging using IVIS Spectrum and IVIS Spectrum CT using the concept of spectral un-mixing) [77].

#### 6.1.1. Disadvantages of FLIT Approach

FLIT imaging constitutes a comparatively high computation time and memory requirements, since it utilizes processing of intensity and depth of incident photons on the CCD (Charge Coupled Device) as well as the physical movement of the motor below the body in the case of trans-illumination imaging (where photons are irradiated from beneath the surface of the mouse). Integration of Computer Tomography (CT) with 3D in vivo imaging consumes even more engineering resources, since it involves a small animal full body X-ray scan followed by 3D reconstruction. If an animal body image is not an essential tool for the study being conducted, non-CT optical devices are used that include a pre-programmed animal atlas with different body orientations.

#### 6.1.2. Advances

The furtherance in photo-thermal therapy and associated agents includes nanoparticles and quantum dots (QDs). They exhibit a large Stoke’s shift which can be irradiated using photothermal sources (lasers with the wavelengths equivalent to the excitation wavelength of the particles) for a longer period of time due to their high resilience to photo-degradation and photo-bleaching [78]. This also involves advancements in chemo-photothermal studies [76]. Integration of 3D in vivo imaging and photo-thermal therapy has paved the way for new buildouts in the field of cancer theranostic imaging and analysis [79,80,81]. Three-dimensional imaging of laser irradiated tumor(s) over a period (time-series study) provides a 360° view of the tumors and their volume variation with time (quantification, tumor volume (mm^3^), and tumor depth (mm)).

Nanoparticles mounted with “antennas” have been synthesized as a platform for computationally guided photo-thermal therapy (PTT) that homogenizes the concept of NIRF imaging and mathematical modeling to improve the efficiency of in vivo PTT [82]. Fluorescence 3D construction has been performed using vector analysis with respect to the tumor and detector coordinates, which is utilized for the calculation of tumor voxel intensities [83]. A recent study by Michele et al. has shown the 3D modelling of tumor spheroids for in vitro analysis [84].

### 6.2. Time Domain NIRF Imaging

Time domain optical imaging is a highly sensitive, low-resolution method. It works on a principle of specimen scanning in a raster modus of 1.0–1.5 mm with a lower spatial resolution. The tissue dependent scattering of the incident and emitted light is also taken into account. Time domain fluorescence imaging is of specific advantage when the sensitivity of an imaging system is particularly limited by high background. The main attribute contributing towards its application is its capability of measuring fluorescence lifetimes of detected signals. Lifetime is defined as the mean time of fluorescence transition, which is the characteristic of each fluorescence molecule and is used to determine signals exclusively derived from specific probes. Imaging studies have reported experiments with non-specific fluorescence, which could be identified, based on the lifetimes of the detected signals [85].

Time-domain imaging has also been successfully employed to selectively subtract background fluorescence from in vivo measurements and also to distinguish signals from two different simultaneously applied NIR fluorophores with similar spectra. Time dependent measurements are employed to increase the accuracy of tomographic reconstructions [86]. It involves estimation of the photons’ time of flights traversing through the tissue to improve image contrast. One such time dependent technique is the frequency-domain photon migration (FDPM approach). FDPM approach involves launching an intensity-modulated excitation light within the tissue and measuring the amplitude-attenuated and phase shifted fluorescence signal. FDPM also effects high signal-to-noise ratio (SNR) by filtering out random ambient light wavelengths. Sevick-Muraca group described the development of a miniaturized FDPM instrumentation to perform small animal 3D optical tomography using trans-illumination geometries, where FDPM and time domain techniques have led to an improved sensitivity in the detection of fluorescent photons originating from deep tissues [87]. FDPM has been reported to have been applied to assess spontaneous mammary carcinoma in animals [88].

## 7. Conclusions

Over the last decade, the field of nanotechnology has experienced tremendous growth and advancement. With substantial efforts by both researchers and the biopharmaceutical industry, a few nanomedicines have already been successfully approved for preclinical and clinical studies. However, the field of nanomedicine is still in its early stages due to unfamiliar types of risks in safety and efficacy that require further discussion and co-operation among researchers and governmental agencies. The challenges in developing NPs for use in MI may be overcome in the near future. NIRF dyes and multimodal probes are expected to broaden their roles in basic cancer research and advance into clinical applications. Taken together, NIRF imaging is promising for early stage cancer detection and cancer therapy, but the development of satisfactory NIRF probes remains challenging for investigators worldwide. Although there are still many hurdles before NIRF imaging can advance to clinical applications, huge opportunities and value exist in this fascinating field. For rapid progress in this field, interdisciplinary collaboration is very much needed.
